# Herd health status and management practices on 16 Irish suckler beef farms

**DOI:** 10.1186/2046-0481-66-21

**Published:** 2013-11-06

**Authors:** James O’Shaughnessy, John F Mee, Michael L Doherty, Paul Crosson, Damien Barrett, Luke O’Grady, Bernadette Earley

**Affiliations:** 1 Animal and Bioscience Research Department, Animal & Grassland Research and Innovation Centre, Teagasc, Grange, Dunsany, Co., Meath, Ireland; 2 School of Veterinary Medicine, UCD, Belfield, Dublin 4, Ireland; 3 Animal and Bioscience Research Department, Animal & Grassland Research and Innovation Centre, Teagasc, Moorepark, Fermoy, Co., Cork, Ireland; 4 Livestock Systems Research Department, Animal & Grassland Research and Innovation Centre, Teagasc, Grange, Dunsany, Co., Meath, Ireland; 5 Department of Agriculture, Food and the Marine (DAFM), Sligo Regional Veterinary Laboratory, Doonally, Co., Sligo, Ireland

**Keywords:** Herd health, Beef suckler, Management practices

## Abstract

**Background:**

There have been few studies published internationally which document herd health management practices in suckler beef herds and no published Irish studies. The study objective was to document herd health status and management practices on sixteen Irish suckler beef herds over a two year period (2009–2010). The farms used in the study were part of the Teagasc BETTER farm beef programme. The mean (s.d.) herd size, stocking rate and farm size was 68 cows (27.6), 2.0 LU/ha (0.3) and 64.3 (21.6) adjusted hectares, respectively. Two questionnaires were designed; 1) a farmer questionnaire to collect information on farm background and current herd health control practices and 2) a veterinary questionnaire to collect information on the extent of animal health advice given by veterinarians to their clients and identification of any on-farm herd health issues.

**Results:**

Dystocia, calf pneumonia, and calf diarrhoea, in that order, were identified as the primary herd health issues in these Irish suckler beef herds. In addition, substantial deficiencies in biosecurity practices were also identified on these farms.

**Conclusions:**

The findings of this study may serve as the focus for future research in animal health management practices in Irish suckler beef herds.

## Background

The effects of herd health problems on the profitability of suckler beef farms are manifested through animal mortality, ill-thrift, cost of treatment, cost of prevention and additional labour [1]. A key component of prevention of herd health problems on suckler beef farms is the identification of those management factors that can significantly impact herd health status. In Ireland, the recorded mortality rates for suckler beef calves at birth and in the first 28 days of life (includes mortality at birth) in 2012 were 5% and 6%, respectively [2]. Considering that a target of 0.95 calves weaned per female mated is the desired production goal [3], then this level of early calf mortality is of concern.

There have been few international and no published Irish studies on herd health management practices and herd health status in suckler beef herds, with previous Irish studies conducted mainly on estimates of disease prevalence [4-6]. Studies performed internationally on suckler beef herds have tended to focus on particular areas of interest, namely, calf health [7-12] or on individual disease conditions [13,14]. Due to differences in production and rearing systems from those practiced in Ireland, predominantly grass-based, adoption of findings from international studies is not always possible or appropriate. The objective of this study was to document herd health status and management practices on 16 Irish suckler beef farms over a two year period (2009–2010).

## Methods

### Farm selection

All sixteen Irish suckler beef farmers voluntarily participating in the Teagasc/Farmers Journal Business, Environment and Technology through Training, Extension and Research (BETTER) beef technology transfer programme were selected for this study. Participants were enrolled in the BETTER farm programme in September 2008. Farmers invited to join the programme were identified based on 3 main selection criteria: 1), farm location (a wide geographic spread was required), 2), a willingness to adopt new farm management practices and 3), a willingness to engage in dissemination activities. However, a further consideration in terms of farm selection was that the farms would be representative of the suckler farming population in terms of financial performance. In this context the benchmark was the gross margin performance (gross output less direct/variable costs of production) of the Teagasc client farmers who completed the Teagasc eProfit Monitor, an internet based financial recording program. Gross margin was selected as the financial measure of most relevance since it best represents the technical efficiency of farm performance (i.e. does not include long term capital costs carried on the farms).

Each farm in the BETTER farm programme was expected to participate in the programme for a minimum period of three years. During that time, farms were visited by the Teagasc agricultural advisors every six to eight weeks.

### Farm details

The farms were located in the south east (n = 4), mid west (n = 3), midlands (n = 3), north west (n = 2), north east (n = 2) and south west (n = 2). The 2010 mean (s.d.) herd size, stocking rate and farm size was 68 cows (27.6), 2.0 LU/ha (0.3) and 64.3 (21.6) adjusted hectares, respectively. Cows of predominantly Limousin, Charolais, Simmental and Belgian Blue genotypes accounted for 50%, 21%, 17% and 5% respectively, of all recorded dam breeds used on these farms over the two year study period. Charolais, Limousin and Belgian Blue were the breeds of sire used in 35%, 29% and 26%, respectively, of all recorded calves born on these farms over the two year study period. Fourteen study farms had both spring (February-April) and autumn/early winter (August-November) calving herds, with the two remaining herds being solely spring and autumn/early winter calving herds, respectively.

The primary system of cattle production varied on these farms. For example in 2009, six farmers sold progeny as weanlings (~ 9 months of age), seven farmers sold finishing cattle for slaughter (~ 18 months of age) and three farmers sold progeny as store cattle (> 12 and < 18 months of age). In 2010, seven farmers sold progeny as weanlings; six farmers sold finishing cattle for slaughter, with three farmers selling progeny as store cattle. All farmers participated in the Animal Welfare, Recording and Breeding Scheme (AWRBS). The main goals of AWRBS [15] are to improve the health and welfare of suckler beef calves by a series of management practices aiming to ease the usually stressful transition from calf to weanling status.

### Farmer questionnaire and farm visits

One farmer questionnaire was used to collect information on herd health management practices employed on these farms, coupled with identifying any herd health problems. Question types were structured to include numerical answers, yes/no answers and the selection of the most appropriate answer from a finite list. Opportunities were also provided for respondents to make general comments. The questionnaire was designed to record information on the following 11 areas: farm background, calving facilities, calving management practices, dystocia/caesarean sections, calf health (birth to weaning), parasite control practices, bull management, management of replacement heifers, trace element and magnesium supplementation, vaccinations and biosecurity. Pilot interviews with two field technicians based at the Animal & Grassland Research and Innovation Centre, Grange were used to check the feasibility and comprehensiveness of the questionnaire. This baseline farmer questionnaire was posted to farmers in advance of farm visits in order to allow farmers to read the questionnaire and to complete some of the basic questions prior to the farm visit. Farms were visited once between January 27th and March 29th 2011, when questionnaires were completed. The average time taken to complete each questionnaire was 2 hours. (Additional file 1: BETTER farm questionnaire).

### Veterinary questionnaire and related information

This questionnaire consisted of 143 questions on the following two areas: 1), extent of animal health management advice, and 2), the veterinarian’s opinion on perceived herd health issues on the farm over the study period. It took on average 45 minutes to complete. When available, the herd veterinarian(s) were visited on the same day and independently of the farm visits in 2011. The herd veterinarian was identified as the veterinary surgeon providing the majority of the clinical service to that farm. If the herd veterinarian was unavailable on the day of the farm visit, the questionnaire was completed by the veterinarian and returned by post, to the project veterinarian (James O’Shaughnessy), at a later date. (Additional file 2: BETTER farm animal health veterinary questionnaire).

Each farmer in the programme acceded to a request allowing access to their Department of Agriculture, Food and the Marine Regional Veterinary Laboratory (RVL) records and access to their Irish Cattle Breeding Federation (ICBF) HerdPlus records. Using the RVL records, information on the results of all post mortem examinations and disease screening profiles were obtained. ICBF HerdPlus records provided information on the following: calving performance records, early calf mortality records and livestock purchases/sales.

### Statistical analysis

Descriptive statistics were used to quantify the various variables in the questionnaires including husbandry and management variables and herd health problems using SAS (version 9.3; SAS Institute, Inc.).

## Results

### Farm financial performance

The gross output value for the two years was considerably greater than either the eProfit Monitor or the average of the Teagasc National Farm Survey (NFS; a representative survey of all suckler farms in Ireland). The financial performance of the BETTER farms over the two year study period is provided in Table 1. The farmers selected for participation in the BETTER farm programme had an average gross margin of €386 per ha in the year prior to joining the programme (2008). This was similar to the eProfit Monitor gross margin for 2008 of €395 per ha. Although variable costs were higher for the BETTER farms, the higher level of output generated resulted in the average gross margin being greater than eProfit Monitor (+50%) or National Farm Survey (NFS) (+395%) farms. Fixed costs were similar on the BETTER farms to eProfit Monitor farms and somewhat greater than NFS farms. The net margin generated on the BETTER farms was much greater than that generated on the eProfit Monitor or NFS farms.

**Table 1:** **Financial performance (€/ha) of the farms participating in the Teagasc/Farmers Journal BETTER farm programme, Teagasc client farmers completing eProfit Monitors (ePM) and NFS**^**1 **^**for cattle rearing farms (2009 and 2010)**^**2**^

	**2009**			**2010**		
	**BETTER farms**	^**3**^**Teagasc ePM**	^**4**^**NFS**	**BETTER farms**	^**5**^**Teagasc ePM**	^**6**^**NFS**
Gross output	1057	849	414	1276	905	446
Variable costs	637	536	306	713	562	305
Gross margin	419	313	108	563	344	141
Fixed costs	470	485	338	467	472	366
Net margin	-40	-172	-230	103	-128	-225

^1^ NFS = National Farm Survey [16,17], representative of national average financial performance for suckler beef farms. ^2^ Financial performance data excludes subsidy and premia payments to farmers from national and European programmes. ^3^ n = 258, ^4^ n = 236, ^5^ n = 304, ^6^ n = 216.

### Farm background

All study farms were conventional (non-organic) enterprises. The majority of study farmers (11/16) had a beef only enterprise, with a minority of farmers (4/16) also having a sheep enterprise. One farmer had a beef and tillage enterprise. Thirteen farmers were fulltime farmers. Ten farmers and nine farmers rented land (i.e. farmers with their own farm who rented additional land) in 2009 and 2010, respectively.

### Calving facilities

Eleven farms used both internal and external calving locations (Table 2). Nine farms had individual maternity pens only. Fifteen farms had concrete floored calving pens. Six farmers cleaned and disinfected calving pens after more than five calvings.

**Table 2:** Calving facilities

						
Calving location	Inside only	5	Outside only	0	Both	11
Calving pen type	Common maternity pen	3	Individual maternity pens	9	Use of both pen types	4
Floor type of calving pen	Concrete	15	Earth	0	Concrete/earth combination	1
Frequency calving pens are cleaned and disinfected	After every calving	4	Between every 2 and 5 calvings	6	After more than five calvings	6

Values are expressed as a number of the total respondents (n = 16).

### Calving management and newborn calf care practices

All farmers responded that they inspected both cows and heifers in stage one of labour once every hour at a minimum (Table 3). Once heifers entered stage 2 of calving (appearance of feet), five farmers only inspected these heifers once every 2–3 hours. Nine farmers stated they used a calving camera as an additional monitoring aid. Fourteen farmers disinfected the umbilicus of newborn calves and all of the farmers used an iodine-based product.

**Table 3:** Calving management and newborn calf care practices

								
Frequency of inspection of heifers/cows instage 1 of labour	Continuous	7	Every 20–30 minutes	6	Every hour	3		
Frequency of inspection of heifers once the feet appear (stage 2)	Every 30 minutes	1	60 minutes	5	90–120 minutes	5	120–180 minutes	5
Frequency of inspection of cows once the feet appear (stage 2)	Every 30 minutes	2	60 minutes	7	90–120 minutes	6	120–180 minutes	1
Length of time newborn calves stay in maternity pens	<1 day	1	1–4 days	12	>4 days	3		

Values are expressed as a number of the total respondents (n = 16).

Three farmers stated that calves spent on average more than 4 days in the calving pens. Twelve farmers did not rely solely on observation of suckling by the calf to ensure adequate colostral intake. To ensure colostral intake, these farmers used varying combinations of observation of suckling, stomach tubing and nipple bottle feeding. Twelve farmers had used external sources of colostrum (natural colostrum sourced off-farm) in the last 5 years.

### Caesarean sections

A majority of farmers (14/16) had caesarean section surgery performed on farm during the study period, with an overall animal-level prevalence of 2% (50/2262) and a range of 0-8% of calved cows per herd.

### Calf health (Birth to Weaning)

Ten and six herds experienced mortalities due to calf pneumonia and calf diarrhoea during the study period, respectively. All six herds that experienced mortalities due to calf diarrhoea also suffered losses due to calf pneumonia. Three farmers used prophylactic treatments to control coccidiosis and five farmers stated that *Cryptosporidium parvum* was diagnosed in the last five years from faecal samples submitted to RVLs.

### Parasite control practices

Twelve farmers treated calves three or more times in their first grazing season for gastrointestinal nematodes, with the remaining four farmers treating their calves twice in the first grazing season.

In both study years, eight farmers turned livestock out to pasture in February. Of the livestock turned out to pasture first, twelve farmers stated that this included livestock less than twelve months of age. Therefore, autumn born calves/weanlings were turned out in the majority of farms early in the grazing season. The majority of farmers (11/16) did not have different parasite control policies for autumn and spring born weanlings. Twelve farmers stated that they generally used an avermectin-based anthelmintic when treating their calves for parasites, with the majority of farmers (10/16) not practicing rotation of anthelmintics.

Nine farmers stated that they had tested cows for liver fluke burden by dung sampling in the study period. All farmers reported treating for liver fluke annually. Fifteen farmers routinely treated all livestock for lice and mange annually, with nine of those farmers using two treatments.

### Bull management

A stock bull was used on twelve study farms, with nine of these farms also using artificial insemination. Four farmers used artificial insemination only. A majority of the farmers that used stock bulls (11/12) always sourced them through purchase, with one farmer both breeding and purchasing stock bulls.

### Management of replacement heifers

Thirteen farmers sourced replacement heifers both from within their own herds and through purchase, while two farmers only used homebred heifers as replacements. One farmer sourced all replacements through purchase only.

### Trace element and magnesium supplementation

Results from the questionnaire indicate that all farmers supplemented both pregnant cows and heifers with a mineral-vitamin mixture for at least four weeks pre-calving, with ten supplementing for a period of up to eight weeks pre-calving. The majority of farmers (15/16) continued to supplement cows post-calving with a mineral-vitamin mixture.

All farmers supplemented cows with magnesium in order to prevent hypomagnesaemia, with the majority (13/16) supplementing in both spring and autumn. The majority of surveyed farmers (11/16) indicated that they only used molasses-based licks/buckets as a method of choice to supplement cows with magnesium.

### Vaccinations

Twelve and eleven farmers vaccinated their breeding stock against BVDV and Leptospira spp, respectively, and three farmers vaccinated their cows against salmonella. No farmers were vaccinating breeding stock against IBR. Twelve farmers vaccinated their calves for clostridial disease. Eight farmers used calf diarrhoea vaccines pre-calving. Seven farmers used respiratory vaccines in calves less than twelve weeks of age.

### Biosecurity

All herds in this study were open; the term open herd being classified as herds where there is any purchase, re-entry and movement of stock. Six farmers indicated that farm staff had regular contact with other livestock. Ten farmers indicated that a foot dip was in use on farm to disinfect footwear of visitors on entering the farm. Four farmers reported that farm visitors (veterinary surgeon, agricultural advisor, artificial inseminator, ultrasound technician) cleaned and disinfected their boots on entering their farm. Eleven farmers indicated that both dogs and cats were kept on farm.

Results from the ICBF stock reconciliation reports indicated that all farmers purchased livestock during the study period. The mean number of purchases per farm for the two years was seventy animals (range 5–407 per herd). Fourteen farmers purchased livestock at 24 months of age and older during the study period. Nine farmers isolated purchased stock on arrival, for indeterminable periods, with only one farmer isolating purchased livestock for a period of thirty days at a distance of at least 3 metres from other livestock on the farm with no indirect contact between purchases and on-farm livestock through either dung or urine. Thirteen farmers tested purchased animals on arrival for the presence of (Bovine Virus Diarrhoea Virus) BVDV antigen. Six farmers indicated that they vaccinated purchased breeding stock on arrival for BVDV. Five farmers indicated that they vaccinated purchased breeding stock for leptospirosis on arrival, with no farmers testing purchases on arrival for exposure to Leptospira spp.

On the majority of farms (9/16), the boundary fence between farm stock and those on neighbouring farms was less than three metres wide. Daily checks to the integrity of the boundary fence (stock-proof) occurred on seven farms, while eight farmers indicated that livestock from adjoining farms entered their lands in the last five years.

Eight farmers indicated that they both cleaned and disinfected livestock trailers if used outside the farm premises. The majority of farms (14/16) had either rivers or streams coursing through their farms. On these farms with watercourses, the majority had farms (12/14) upstream/upriver from their farms, with livestock on nine farms having access to these watercourses.

Eight farmers stated that both sheep and cattle farms adjoined their farms. Four farmers stated that only cattle farms adjoined their land while another four farmers stated that cattle, sheep and tillage farms adjoined their farm.

### Veterinary questionnaire

**Figure 1:**
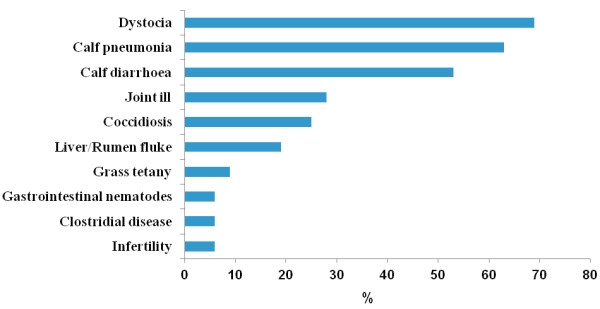
Health problems (% of 32 herd-years in 2009 and 2010) identified on 16 suckler beef farms by veterinary practitioners in the veterinary questionnaire.

Results of the veterinary questionnaire (Figure 1) indicate that veterinarians identified dystocia, calf pneumonia and calf diarrhoea, in that order, to be the most significant herd health problems in both study years on these farms. Coccidiosis and joint ill were also regarded as major health problems.

**Figure 2:**
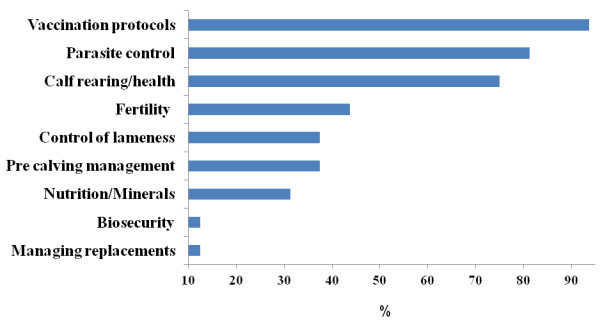
Veterinary advice given to 16 suckler beef farmers (% of 32 herd-years in 2009 and 2010).

The majority of surveyed veterinarians (15/16) indicated that they regularly advised their clients on vaccination protocols (Figure 2). Thirteen veterinarians regularly advised their clients on parasite control while twelve veterinarians advised their clients on calf rearing/health. A minority of veterinarians (2/16) advised their clients on biosecurity.

### Animal health results

Thirty calf carcases were submitted to the RVLs for post mortem from nine farms (range 1–7 per herd submitting) during the study period. Calf pneumonia, calf pneumonia/enteritis complex and calf enteritis, in that order, were identified as causes of death in twelve, four and three calves, respectively. The cause of death could not be determined in another six calves although two of these calves had low zinc sulphate turbidity test results (2 and 7 days old; 1 ZST unit, respectively). Of the remaining calves submitted for post mortem, two calves died from septicaemia, one calf developed peritonitis, one calf developed pericarditis, one foetus inhaled meconium and one calf died due to intestinal volvulus. Fifteen faecal samples from calves (< 1 month old) with diarrhoea from six farms were submitted for examination to the RVLs. *Cryptosporidium parvum* was identified in six samples from four farms while rotavirus was identified in four samples from three farms.

Faecal samples were collected from seventy two cattle from nine farms by veterinarians to determine their liver and rumen fluke burdens. Rumen fluke eggs were identified in twenty samples submitted from six farms while liver fluke eggs were detected in four samples from three farms.

Results from the ICBF HerdPlus records indicate that the overall incidence of calving assistance and dystocia over the study period was 22.5% and 5.8%, respectively. The incidence in primiparae for calving assistance and dystocia was 33.3% and 8.6%, respectively. The incidence in pluriparae for calving assistance and dystocia was 20% and 5%, respectively. Data for calf mortality, calving interval and calf output per female are presented in Table 4.

**Table 4:** Fertility and calf mortality on 16 suckler beef herds

	**Total no. of calvings**	**Calving interval (days)**	**Mortality-dead at birth (%)**^**1**^	**Mortality-dead at 28 days (%)**^**2**^	**Calves per cow per year**^**3**^
2009	1086	375 (14.5)	2.36 (1.75)	3.81 (2.59)	0.84 (0.08)
		(353-413)^4^	(0%–5.9%)^5^	(0%–8.3%)^5^	(0.63–0.98)^4^
2010	1176	382 (16.3)	1.3 (1.58)	4.25 (3.53)	0.82 (0.1)
		(356–421)^4^	(0%–4.9%)^5^	(0%–10.5%)^5^	(0.5–0.97)^4^

Values are expressed as a mean (S.D.) of total animals.

^1^ Number of calves born dead as a proportion of all births during this period, ^2^ Number of calves born dead or dead within 28 days, as a proportion of all births recorded during this period, ^3^ Number of calves per cow per year, expressed as a proportion of all eligible females in the herd, eligible females being females equal or greater than 22 months of age. ^4^ Herd range in mean value. ^5^ Range among herds.

Source: ICBF Herdplus. (http://www.icbf.com/services/herdplus/beef/index.php).

## Discussion

This is the first study to document animal health management practices and herd health status in Irish suckler beef herds. Veterinarians regarded dystocia, calf pneumonia and calf diarrhoea, in that order, as the primary herd health concerns in these Irish suckler beef herds. However, responses from the first farmer questionnaire also highlight the inadequate biosecurity practices employed on these farms.

The incidence of caesareans in the present study was higher than previously reported international studies [18-21]. The use of dams and sires of predominantly continental genotypes on these farms may have been a contributory factor to the recorded prevalence of caesarean surgeries performed on these farms. There was a 19% greater use of Belgian Blue as a sire in these herds than what is used in the national suckler beef cow population [22]. This is likely a reflection of the practice on some farms where weanlings are exported, with those markets paying a premium for superior carcass conformation. In addition, of the cows with a recorded calving ease score of four [23,24] Belgian Blue was the breed of sire used in sixty per cent of those calvings. Previous studies have shown the greater incidences of dystocia that can occur when using continental sires as opposed to traditional British sire breeds [25].

On seven farms heifers/cows were continuously inspected in stage one of labour. While somewhat surprising, it is the opinion of the project veterinarian (James O’Shaughnessy) that the responses given accurately reflected management practices. As all farms had seasonal calving patterns, it is likely that during the calving season heifers and/or cows would regularly be at different stages of the birthing process in the calving sheds and as a result they might be observed more frequently than otherwise might be expected. The increased frequency of observations of heifers/cows on some farms would also allow farmers to make more informed decisions on when to intervene in stage two of labour as they could determine the level of progress over a defined period of time. Five farmers only inspected heifers once every 2–3 hours in stage 2 of labour. This practice of inspecting heifers once every 2–3 hours in stage 2 of labour can potentially have a significant impact on rates of perinatal mortality [26], given the higher incidences of dystocia in heifers [20,21].

The finding that both calf pneumonia and calf diarrhoea were the two most significant causes of mortality/morbidity in the present study is in agreement with work carried out in suckler beef herds [19] and in dairy herds [27,28].

Results from the farmer questionnaire on calving facilities (Table 2) show that on six farms, calving pens are only cleaned and disinfected after more than five calvings. This practice of infrequent cleaning and disinfection of calving pens on some farms is a likely contributory factor to neonatal disease in these herds [12]. The extended periods of calf residency in calving pens on some farms is an additional concern. Individual maternity pens, by design are restricted in space. Therefore, the longer a calf resides in a maternity pen, which is not subject to frequent cleaning and disinfection, the greater the risk of infection.

The majority of farmers (11/16) used both internal and external calving locations. Each location has its own benefits. Despite the benefits of reduced pathogen build up in calving animals outdoors [29], the use of a designated calving site, internally in this instance, allows for better management of calvings and quicker resolution of calving difficulties, should they arise.

The majority of farmers (9/16) had individual maternity pens only. The neonatal health benefits of using individual versus common maternity pens are largely inconclusive [27,30,31]; however, herd size appears to be an important factor [30]. However, ensuring calving pens are regularly cleaned and disinfected is a prerequisite in the prevention of neonatal disease [12].

Joint ill was also identified by veterinarians as a significant health problem on some study farms. This is a reflection of the inadequate neonatal health management practices employed on these farms. Deficiencies in some or all of the following are possible contributing factors: 1), ensuring calves receive adequate volumes of colostrum as soon as possible after calving, 2), calves born into and residing in a hygienic environment, and 3), dressing the calf’s navel with an appropriate antiseptic solution. Respiratory acidosis is also a consideration as a calf’s ability to absorb immunoglobulins may be impaired as a consequence of dystocia [32].

The risk of introducing disease into these herds was high as 14 farmers sourced replacement heifers through purchase and 12 farms purchased their stock bulls. The practice of purchasing adult stock greatly increases the biosecurity risk given the increased exposure time these adult animals will have had to various pathogens.

All farmers who tested purchased livestock for the presence of BVDV did so as a result of programme participation. This also occurred before the voluntary phase of the national eradication programme [33]. Considering the currently reported prevalence of BVD antigen-positive cattle in an Irish cattle population [34] then the testing for BVD antigen in purchased livestock on these farms was prudent. Farmers in the present study only tested purchases for BVD antigen, with no attempt being made to determine the status of purchases for diseases such as IBR, leptospirosis or Johne’s disease. Although there is significant interest at present (2013) among the Irish farming population in BVDV, due to the national eradication programme, greater attention also needs to be focused on testing purchased stock for other important biosecure diseases. Given the previously reported seroprevalence in Irish herds for *Mycobacterium avium* subspecies *paratuberculosis* (MAP) [4], IBR [5] and *leptospira interrogans*[6], then a knowledge of the seller’s herd health history coupled with determining the serological status of purchased stock to these diseases should be considered.

The use of leptospirosis vaccines by five farmers on purchased stock of unknown infection status is a beneficial practice but consideration should also be given to using a combination of both immunisation and antibiotic treatment for all purchased cattle on arrival at the farm [35], to eliminate renal shedding of leptospires and prevent re-colonisation.

The practice on six farms where staff had regular contact with other livestock lends itself to the possibility that farm staff could act as vectors of disease on these farms. The fact that only on a minority of farms (4/16) visitors cleaned and disinfected their boots on entering the farms, further serves to exacerbate the biosecurity risk. This highlights the lax attitude towards biosecurity that existed on majority of these study farms.

Additionally, with the majority of farms not having a 3 metre divide between their stock and the stock of neighbouring farmers, a biosecurity risk from direct animal to animal transmission of disease exists. Although a three metre gap between the boundary fences of neighbouring farms is a well established biosecurity standard, it may however be ineffective against the transmission of viruses such as bovine herpesvirus 1 [36] or BVDV.

The frequency of dosing of calves in their first grazing season was considerably higher than what has previously been reported in beef herds [37], where less than 25% of beef calves were treated three or more times in their first grazing season. As milk accounts for more than 80% of the diet of suckler calves in the first 3 months of life [38] and considering that on half of these farms animals were turned out to pasture in March and April when pasture larval burdens are starting to decline, then the frequency of dosing in the first grazing season appears quite high. In addition, faecal egg counts of suckler cows tend to be low [39-41], thus further diminishing the possibility that these spring born calves acquired worm burdens of sufficient quantity so as to impair thrive.

The fact that a majority of farmers did not have different parasite control strategies for autumn and spring calves/weanlings implies a lack of appreciation for the epidemiological differences that exist between these two age groups in relation to parasite control. Autumn born calves are more susceptible to gastrointestinal parasites, especially if turned out to grass earlier in the grazing due to higher herbage intakes and earlier weaning dates when larval burdens on pasture are more abundant.

The dosing strategies employed on these farms, where the majority of farmers used an avermectin-based anthelmintic on calves and did not rotate anthelmintics, may, in the long term, lead to anthelmintic resistance [42].

A majority of farmers had faecal sampled cows over the study period to determine liver fluke burdens. This practice allows farmers to make more informed judgements on whether or not to treat their cows for liver fluke. However, given the fluctuations that can occur in fluke faecal egg output over a 24 hour period [43] and considering the sensitivity of dung sampling ranges from 66-69% [44,45], additional tools should be considered to aid estimation of fluke burdens on these farms. These include previous farm history of fasciolosis, grazing location and use of meteorological data such as rainfall amount and temperature.

The number of farmers treating cows for liver fluke annually here (16/16) differs from that in a study carried out in south west England on beef herds, 79% of which were beef suckler herds [37]. In that study, only 35% of farmers treated specifically for liver fluke. The high levels of rainfall experienced, particularly in the summer of 2009 [46], are likely to have contributed to the challenge posed by both liver fluke and rumen fluke on these farms.

Given that gastrointestinal parasites and lungworm were not perceived by veterinarians on these study farms to be significant health issues may further serves to highlight the frequent nature of anthelmintic treatments employed on these farms.

The identification of rumen fluke eggs from faecal samples on six study farms over the study period is not surprising as clinical signs of rumen fluke infection have been increasingly diagnosed in recent years in Ireland [47].

All farmers supplemented pregnant cows and heifers pre-calving with trace elements, with fifteen farmers also supplementing post-calving. This reflects awareness among the farming population in the trace element status of Irish cattle. A study carried out in Ireland on the trace element status of dairy and suckler cows [48], found a higher proportion of suckler cows deficient in trace elements compared with dairy cows, with a greater proportion of suckler cows being deficient in iodine and selenium in the autumn as opposed to the spring. In a retrospective study on French and Belgian dairy and beef herds [49], a number of significant associations were found in the beef herds between trace element deficiencies and herd health problems. Deficiencies in copper, zinc or selenium were associated with increased risk of calf diarrhoea, perinatal mortality and poor growth. Therefore the practice of supplementing the diet of pre-calving cows and heifers in this study is warranted, considering the deficiencies that may exist and the herd health problems that may arise as a result of those deficiencies.

The number of farmers that supplemented cows with magnesium was higher than reported previously in another Irish study where only 79% of suckler farmers supplemented their cows to prevent hypomagnesaemia [50]. The practice of providing sources of magnesium to cows in the autumn is also a necessary health management practice considering the results of a previous study where, on average, 30% of suckler cows had serum magnesium concentrations classified as either deficient or marginal [51]. Serum magnesium concentrations are reliant on dietary intake owing to lack of availability from body stores and are therefore very sensitive to external stressors interfering with intake such as inclement weather or feed availability. In an Irish study [50], the use of molasses-based licks/blocks as a method of supplementation was found to be unreliable in reducing the incidence of hypomagnesaemia. This may be as a result of cows ingesting insufficient quantities of magnesium during risk periods, namely spring and autumn. In addition, the content of molasses in these licks/blocks will also influence intake of magnesium as the content of molasses is directly related to palatability of this form of supplementation. The reliance on molassed-based licks or buckets as the sole form of magnesium supplementation on the majority of these farms (11/16) is thus, a cause of concern.

The number of farmers that vaccinated their breeding stock was higher than previously published international studies in beef herds for both BVDV [52] and leptospira spp [53]. These results are not surprising given the fact that a large majority of surveyed veterinarians (15/16) routinely advised their clients on vaccination protocols. In addition, both participation in the programme and the recent widespread media exposure in Ireland on the deleterious effects of BVDV on both herd health and fertility in the farming press may have been additional contributing factors. The number of farmers that vaccinated their breeding stock for BVD is comparable to the percentage of farmers vaccinating their breeding stock for BVDV in Irish dairy herds [54].

The overall mean calving intervals in these herds was considerably less than the national calving interval of 406 days for beef suckler herds [2] and is probably partially explained by the regular interaction between these farmers and their agricultural advisors/veterinarians. The cause of the increase in the mean calving interval from 375 days in 2009 to 382 days in 2010 (Table 4) is unknown.

Although figures quoted for perinatal mortality rates often encapsulate different time periods [7,25], the results here however, compare favourably with those studies and with the national average of 5% [2]. The impact of dystocia on perinatal mortality rates has previously been shown [55].

The percentage of calves born dead or dying within the first 28 days of life in this study is comparable with the national average of 6.12% [2]. The increase in mortality (0-28d) from 2009 to 2010 serves to highlight the constant challenge that neonatal health management presents.

The results for calf output of breeding females in these herds (0.84 in 2009 and 0.82 in 2010) was higher than the national average of 0.78 [2]. However, performance in these herds is still inadequate when compared to the target of 0.95 calves weaned per breeding female mated [3].

## Conclusions

Results from both the farmer and veterinary questionnaires indicate that dystocia, calf pneumonia and calf diarrhoea were the main herd health problems in these suckler beef herds. Responses from both questionnaires also indicate that biosecurity practices were suboptimal.

The extensive use of sires of continental origin, particularly Belgian Blue, is likely to have contributed to the high incidence of caesarean sections/dystocia in these suckler beef herds.

The infrequent cleaning and disinfection of calving pens on some farms, coupled with the potential for extended periods of residency of calves in those same pens, may have contributed to the incidence of neonatal disease on these farms. The reliance on observation of suckling as the only method of ensuring adequate colostral intake on four farms is an additional risk factor for neonatal disease.

The biosecurity practices employed on these farms, whereby BVDV was the only biosecure disease tested for in purchased livestock on most, but not all farms, reflects deficiencies in biosecurity practices on these farms. This is also reflected in responses to the veterinary questionnaire, where only a small percentage of veterinarians advised their clients on biosecurity. The herd veterinarians to these farms need to play a greater role in ensuring that the risk of introducing disease onto these farms is minimised.

The findings of this study may serve as the focus for future research in animal health management practices in Irish suckler beef herds.

## Competing interests

The authors declare that they have no competing interests.

## Authors’ contributions

BE, PC, JM, MLD, DB designed the study. JO’S developed the veterinary and farmer questionnaires which underwent an extensive review process by all authors; JO’S collected all the data; LO’G advised on data analysis; JO’S and BE analysed the data; JO’S prepared the paper. All authors read, critiqued and approved the final manuscript.

## Supplementary Material

Additional file 1 BETTER farm animal health questionnaire. Click here for file

Additional file 2 BETTER farm animal health veterinary questionnaire. Click here for file
