# Supplementary material for: Herd health status and management practices on 16 Irish suckler beef farms
Source: Ir Vet J. 2013 Nov 6;66(1):21. doi: 10.1186/2046-0481-66-21 (PMC3903451; doi:10.1186/2046-0481-66-21)
Supplement: Additional file 1 — BETTER farm animal health questionnaire. [file 2046-0481-66-21-S1.pdf]

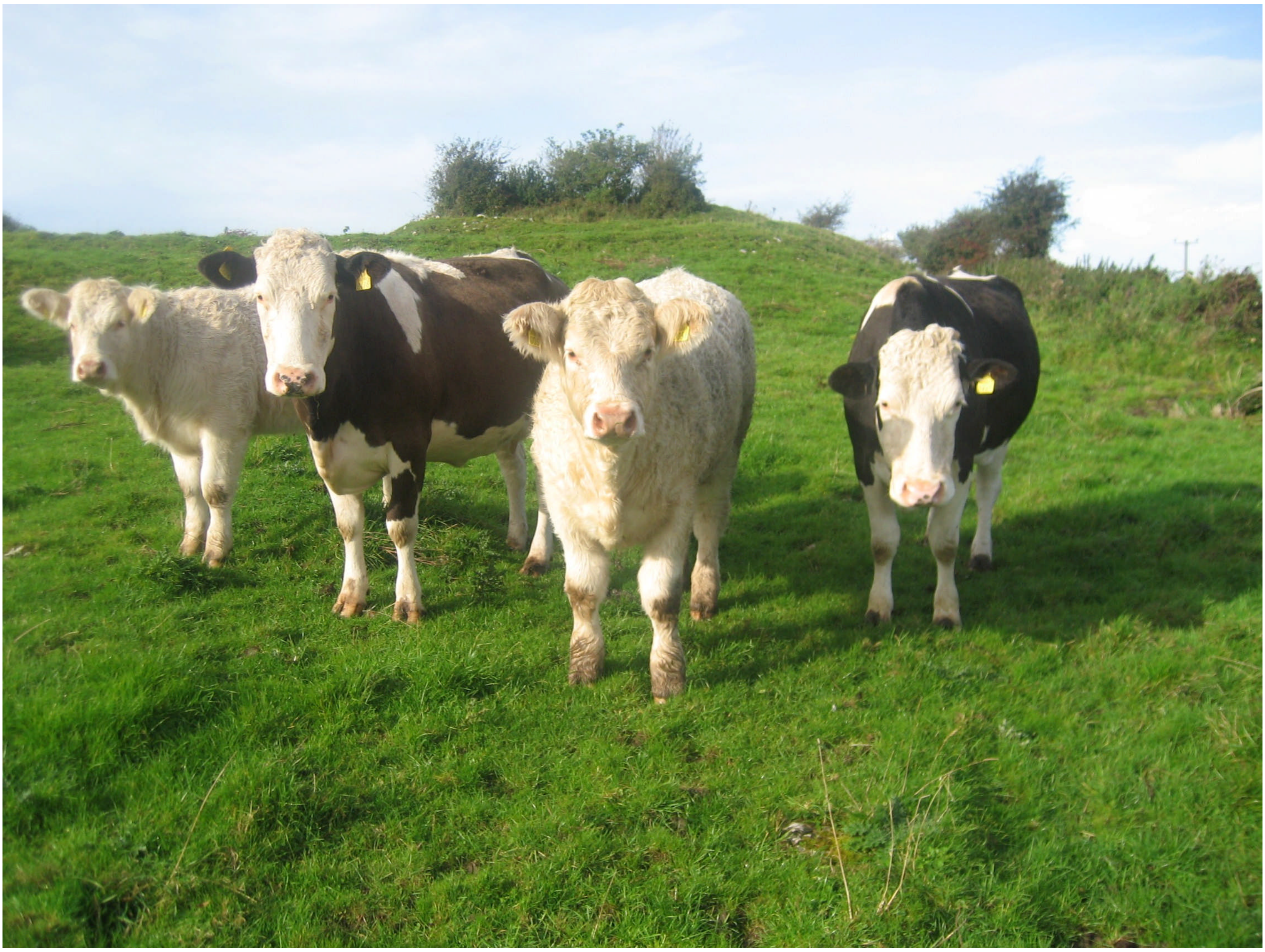

BETTER farm project animal health background information

Farmer name:

Address:

Phone number: Mobile:

E-mail address: GPS grid code:

ICBF Log in information:

Herd number:

Teagasc advisor:

Phone number of advisor:

Veterinary clinician:

Veterinary practice address:

Phone number of clinician: mobile:

Enterprise type:

Beef	Sheep	Dairy	Tillage	Other

Organic farm:

Yes		No	

Is the farmer in the suckler welfare scheme?

Yes	
No	

Livestock numbers:

	2009	2010
Total number of livestock		
Young (<12 months)		
12-24 months old females		
12-24 months old males		
Females over 24 months		
Males over 24 months		

Stocking density:

2009		2010	

Calving and fertility data:

- calving pattern in 2009 and 2010

	2009	2010
Spring		
Autumn		
All year round		

- Number calved per month 2009/2010

	Jan	Feb	Mar	Apr	May	Jun	July	Aug	Sep	Oct	Nov	Dec
2009												
2010												

- Total number of calvings in 2009 ________ 2010 ____________
- categories of calving based on ICBF events sheets

	normal	Some assistance	Considerable assistance	Vet. assisted
	1	2	3	4
Amount				
2009				
2010				

- calving interval 2009 ______ 2010 _________
- number of calves per cow/per year 2009 ____ 2010 _____
- number of calves born dead

  2009 _________ 2010 ___________

- number of calves born dead or dead within 28 days

  2009 _______ 2010 _____________

BETTER farm animal health questionnaire

Date:

Farmer name:

- Background to farm
- Housing and calving facilities
- Calf/Yearling health (0-12 months old)
- Calving time
- Parasites/worming
- Cow health
- Lameness
- Nutrition
- Fertility and breeding
- Biosecurity

Background to farm:

(1) Part (pt) or full time (ft) farmer:

Pt		Ft	

(2) If you are a part time farmer, was your source of off farm work seasonal or continuous in 2009 and 2010?

	2009	2010
Continuous		
Seasonal		

(3) If seasonal then for how many months? ________________________

(4) What months of the year did this involve? ____________________

(5) Size of farm

Hectares

(6) How much of the farm was owned/ rented in 2009/2010?

	2009	2010
Owned		
Rented		

(7) Type of herd:

		Definition
Closed herd		No animal re-entry, no bought in animals, no fence line contact with other herds.
Restricted herd		Only re-entry of existing animals e.g. from shows
Open herd		Routine purchase, re-entry and movement of animals; direct co-mingling of introduced animals of unknown background with herd animals

(8) If the herd is open how many animals were bought in 2009 and 2010?

	2009	2010
Young (<12 months)		
12-24 months old females		
12-24 months old males		
Females over 24 months		
Males over 24 months		
Total number of livestock bought		

(9) How many weanlings produced on farm in 2009 and 2010?

2009		2010	

(10)What happened to weanlings produced on the farm in 2009 and 2010?

	2009(No.)	2010 (No.)
All weaned and sold		
Some but not all sold		
Sold as stores		
Finished on farm		

(11) Did you have store cattle (12-24 months old and not for breeding) in 2009/2010?

	Yes	No
2009		
2010		

(12) How many in each year?

	Number
2009	
2010	

(13)How many of these were finished on farm in 2009 and 2010?

	heifers	Steers	Bulls
2009			
2010			

(14) What age were these animals finished at?

	Heifers	Steers	Bulls
2009			
2010			

(15) In 2009 and 2010 how many females 22 month of age and over were not intended to be used for breeding? (any female 22 months or older is classified as a breeding animal by the ICBF).

2009	
2010	

Housing and calving facilities:

(1a)Where did the cows, calves and other livestock overwinter in 2008/2009? Quantify.

	Cows	Calves	Bull(s)	Steers	Heifers	Sheep
Slatted housing						
Slatted housing with rubber mats						
Creep area						
Straw bedded						
Cubicle housing						
Peat based						
Woodchip based						
Outdoor pad						
Individual pen						
Grazing winter brassicas e.g. kale						
Other						

(1b)Where did the cows, calves and other livestock overwinter in 2009/2010? Quantify.

	Cows	Calves	Bull(s)	Steers	Heifers	Sheep
Slatted housing						
Slatted housing with rubber mats						
Creep area						
Straw bedded						
Cubicle housing						
Peat based						
Woodchip based						
Outdoor pad						
Individual pen						
Grazing winter brassicas e.g. kale						
Other						

2) If the calves have access to a creep area how many are usually in there? ___________

3) Is there separate accommodation for a bull? ____________________________

4) Is there an isolation box/area for sick animals? _____________

5) Is there an isolation box/area for bought-in animals? ___________

6) Do animals calve inside or outdoors ?

Calve indoors	
Calve outdoors	
Both	

7) What facilities exist?

Common calving pen	
Individual pens and how many (1 per 20)?	
If none of the above, please specify	

8) What is the floor of the calving pen made of?

Concrete	
Earth	
If none of the above, please state	

9) How long do newborn calves stay in the calving pen until they join the rest of the calves? ______________

10) How often are calving pens cleaned and disinfected?

After every calving	
Between every 2 and 5 calvings	
After more than 5 calvings	

11) What disinfectant is used to clean the calving pen? _____________________

12) Do you follow manufacturer's instructions when using it? _________________

13) How often is bedding material added to the calving pens?

Every day	
After every calving	
Between 2 and 5 calvings	
After more than 5 calvings	

14) What bedding material is used? ___________________________

Calf /Yearling health (0-12 months):

1) Of the calves that died from birth to weaning what were these deaths attributed to? In the boxes provided please enter the number and age of the animals that died.

	2009	2010
Stillborn or weak calves		
Diarrhoea		
Pneumonia		
Septicaemia		
Accidental deaths/Injury		
Meningitis		
CCN		
Congenital defects		
Joint ill/navel ill complications		
Bleeding calf syndrome		
Clostridial disease		
Coccidiosis		
Hypomag. tetany		
Other reasons		

2) Have you had any calves born in the last 5 years with congenital defects and if so what was the diagnosis? ____

3) Are dams vaccinated with a pre-calving scour vaccine? _____________

4) What is the product if one is used? ______________________

5) Do you use any respiratory vaccines on the calves in the first 12 weeks of life? _________________________________

6) If you do what product do you use? _______________

7) How many doses? _____________

8) Are calves vaccinated against pneumonia pre-housing/pre-sale? Please specify

____

9) If so when is it done in relation to sale or housing? _______________

10) What product is used? ________________________

11) How many doses are used per animal? __________________________

12) Do you use any preventative medicine for coccidiosis? ________________

13) If you do, what product do you use? __________________

14) When do you give it? ______________________

15) What age do you give it at? _________________________

16) Has Cryptosporidium been diagnosed on your farm in the last five years? _____

17) If it was diagnosed do you now use any preventative medicine for it? _________

18) What product do you use? __________________

19) What age do you first give it at? _____________

20) How much do you give per day? ______________

21) How long for? ___________

22) Do you vaccinate your calves against Clostridial diseases such as blackleg for example? ____________________________

23) What product do you use? ________________

24) How many doses do you give? ________________

25) When do you give it? _______________________________________

26) Have any of your calves suffered from ringworm in 2009 or 2010?

2009	
2010	

27) What treatment did you undertake for it? __________________________

____

28) Are calves creep fed before weaning? ______________________

30) How long are they creep fed before weaning? _____________

31) How long are they fed concentrates after weaning? __________

32) How much creep is given per head? _______________________

33) Do you forward creep graze (allow calves graze ahead of cows) ? ________

34) What age are calves weaned at? _____________

35) What age are calves disbudded at? ____________

36) Do you use local anaesthetic at any stage? _____________________

37) Are male calves castrated? ______________

38) What age is it performed at? ___________

39) What method is used? _______________

40) Are cows with female calves separated from cows with male calves at pasture? _______________________

41) At what age is it done at? ____________________________________

Calving time:

1) Number of vet-assisted calvings in 2009 and 2010

2009 ___________ 2010 _____________

2) Number of calvings that were caesareans? 2009______________2010 ___________

3) How often are animals inspected once they are sick to calve/stage 1 of labour has begun? ___________

4) How long do you leave them to calve unassisted once the feet appear?

Heifers _____________ Cows ____________________

5) Do you use any aids such as a calving camera? _______________

6) Are navels disinfected? ______________?

7) If so what is used? ________________

8) Are afterbirths disposed of? ______________

9) Where are they disposed of? _____________

10) Do you ensure the calf has received colostrum by ......

Observation of suckling		
Stomach tubing		
Bottle and teat		
Other method and specify		

11) Do calves receive colostrum only from their mothers? ________________

12) Are any calves supplemented with additional colostrum that you may have stored from other on farm cows/heifers? _______________

13) Have you ever used external sources of colostrum in the last 5 years ? ______________

14) If you do where do you get it? ________________________

15) Do you know the health status of that herd? _____________

16) For spring born calves how soon after calving is the cow and calf turned out to grass? ______________________

Parasites/worming:

(1) How often are calves wormed in their first grazing season?

Once	
Twice	
Three times	
More than this	

2) What products are used? ______________________

3) When are they given? ____

4) When are they given in relation to either sale/housing? ____________________

5) Do you rotate wormers over the course of the season? _________________________

6) In terms of parasite monitoring during the grazing season, are growth rates measured and used as a guide for parasite burden? ______________________

7) Are dung samples collected for egg counts to measure parasite burden? ________________________

8) Is there a separate worming policy for spring and autumn born calves?

Yes		No	

9) If there is please specify?

10) Is there a worming control plan is in place for second season grazers (stores)?

Yes		No	

11) How many times are they dosed? ________________

12) What are they dosed with? ________________________

13) When do they receive it in relation to turnout? ______________

14) When do they receive it in relation to housing? _________________

15) Are cows ever tested/ examined for liver fluke?

	By dung sample	At the abattoir
Yes		
No		

16) When was this done? _______________

17) Has rumen fluke ever been diagnosed on your farm either by faecal analysis or by post mortem? ______________________

18) If so when was it diagnosed? __________

19) What treatment regimen was put in place? _______________________________

20) Are cows treated for liver fluke annually? ___________________________

21) What stage of the year are they treated? ______________________

22) What product is used? ______________________

23) Do they receive a second fluke dose if they are outwintered? ____________

24) If they do when do they get it in relation to the first dose? ________________

25) Are all livestock treated for lice/mange annually? _________________

26) How many times are they treated? ________________________

27) When exactly are they treated? ______________________________

28) What product is used? ________________________

Cow health:

1) Do cows receive pre-calving minerals? ______________________

2) What mineral mix is used? ________________________

3) How long is it given for? ____________________

4) How much is used? ________________

5) How is it given?

Mineral lick	
Bolus	
Granulated on silage/meal	
In the water supply	
Other method	

6) When was the herd last blood sampled for trace elements/minerals? ___________

7) What was screened for?

Copper	
Selenium	
Iodine	
Other	

8) How many animals were used for the sampling? ___________________

9) If applicable what control plan was put in place?

10) Are all cows, breeding heifers and breeding bulls vaccinated for any of the following?

	Tick if used	Vaccine product	No. of doses given	Time of year given	Cows(no.)	Bulls(no.)	Heifers(no.)
BVDV							
Lepto.							
IBR							
Salmonella							
Clostridia							
Other							

11) Are cows supplemented to prevent for grass tetany? ____

12) Which of the following methods do you use?

Bolus	
Pasture dusting	
In water	
Licks	
High mag. nuts	
Other	

13) For the method chosen above what is the product used?

____

14) How many of the cows are you using this treatment on? ______________

15) How much of the product is used? _________________

16) When is it given? __________________________________

17) If applicable, do cows that are dried off over the summer receive dry cow tubes?

Yes	
No	

18) Are cows dried off in batches or all dried off together?

19) Is there any fly control over the summer months? ________________

20) If there is which one of the following best describes your fly control policy.

Application of pour-on	
Stockholm tar	
Insecticidal ear tags	
Use of teat tape	
Regular topping of fields	
Resisting drying off cows between july-sept.	
Keeping animals away from ponds/water courses	
Keeping animals away from wooded areas	
Other method	

21) For any fly control products please state the product and how often it's applied.

22) Has there been a reported/recorded case of Johne's disease in the last 5 years on the farm? _________________

23) If there was, was a control plan put in place? ___________________________

24) Has there been any confirmed cases of Salmonella on your farm in the last 5 years? ______________________

25) Has there been any on farm cow deaths in the last 5 years where Clostridial disease was confirmed as the cause? _______________________________

26) In total how many cows died on farm in 2009 and 2010?

2009		2010	

27) Were post mortem examinations carried out? _____________________

28) What causes were identified?

Lameness:

1) Are cows/bull(s) routinely foot trimmed?

Yes		No	

2) If they are what time of the year is it done? ____________________________

3) Who performs it? ____

4) How many times was it carried out in 2009 and 2010?

2009		2010	

5) Do you use a footbath on farm? ____________________

6) How often is it used? __________________________________

7) When do you use a footbath (time of year)? _______________________

8) What type of footbath solution is used (+concentration)?
____

9) How often is it changed/ How many cow passes before it's changed?
____

10) Have you had lameness issues on your farm in 2009 and 2010?

2009		2010	

11) What time of year did it occur? __________________________________

12) If you had what was diagnosed as the cause?
____

13) Who diagnosed the problem? _______________________________________

14) How many animals were involved?
____

15) What was done about it?
____
____

Nutrition:

(1) Are cows condition scored?

Yes	
No	

(2) If yes, how many times were they done in 2009 and 2010?

2009	
2010	

(3) When was it performed in 2009 and 2010?

	Pre-breeding	Drying-off	Other (specify)
2009			
2010			

(4) By whom? ________________

(5) What combination of the following are cows fed over the winter period?

Pit silage	
Round bale silage	
Hay	
Straw	
Concentrates	
Outwintered on brassicas e.g. kale	
Other	

(6) Did you buy in feed supplements in 2009 or 2010?

Yes		No	

(7) If so what did you buy in?

	2009	2010
Silage		
Concentrates as nuts		
Hay		
Straights		
By products like brewers grains etc (specify please)		
Straw		

(8) Do you use a diet feeder? ________________

(9) What is the composition of the diet?

(10) Is the silage routinely analysed? ________________________

(11) If it's analysed do you make changes to the winter diet as a result, please specify?

____

(12) Apart from your Teagasc adviser do you use a nutritionist? ________________

(13) Do cows receive minerals post calving? ______________________

(14) How are they given?

Mineral lick	
Bolus	
Granulated on silage/meal	
In the water supply	
Other method	

(15) What is used? ________________

(16) How long for? ____

(17) What is the grazing system at pasture?

Continuous	
Strip	
Rotation	
Other	

(18) Do you budget for grass availability? _____________

(19) If so which of the following guides do you use?

Visual	
Sward height	
Quadrant and shears	
Plate meter	

(20) If not what do you use as a guide? _______________________

(21) In times of shortage do you supplement the cows? ____________________

(22) What do you supplement with? ____________________________

(23) How much do you give? ____

(24) In the breeding season and for the 2 months before it could you describe the diet of the cows.

(25) What is the diet of the the cows in the 6 weeks before calving?

(26) Where are they in this period?

____

(27) What were your turnout dates in 2009 and 2010?

2009	
2010	

(28) Which category of animals get turned out first?

	2009	2010
Young (<12 months)		
12-24 months old females		
12-24 months old males		
Females over 24 months		
Males over 24 months		

(29) What was your housing date for 2009 and 2010?

____

Fertility and breeding:

1) How many females were submitted for breeding in 2009/2010?

2009		2010	

2) How many of these were heifers?

2009		2010	

3) How many females aborted in 2009 and 2010?

2009		2010	

4) Were samples sent to the laboratory? _____________

5) What was identified? ____

6) What control measures were subsequently put in place?

7) Has there been any diagnosed cases of mucosal disease in the last 5 years? ________

8) Has the herd ever been screened for BVDV before 2010? _______________

9) Is the herd routinely monitored for BVDV? _________________

10) If this is done how is it performed?

	Specify age
Ear notch testing	
Blood testing	

11) What was the outcome? Were PI's (carriers) identified?

12) If carriers were identified what did you do with them?

Nothing	
Euthanized immediately	
Isolated and fattened them	
Sold at auction	
Slaughtered immediately	
Other	

13) Have you ever seen the AHI BVD leaflet? ____________________

14) Have you found it helpful? ____

15) Did you use its information for screening/monitoring? ________________

16) Have you had any problems with Leptospirosis (confirmed by lab.) in the last 5 years?

____

17) Has Neospora ever been diagnosed on the farm as a cause of abortion? ________________________

18) If there has been, is there any on farm policy for testing future breeding females? ___________________________________

19) What is your source of semen?

Stock bull		A.I.		Both	

20) If you use stock bulls then how many do you have? ________________

21) What is the usual source of your stock bull?

Auction	
Home bred	
Private sale	
Other	

22) If you buy in a bull, how soon before the breeding season is this done?

____

23) What age do you normally buy a bull at? _______________

24) If you have more than one stock bull do you rotate them with the breeding herd?

____

25) At any one time what is the bull/cow ratio in the breeding season? ______________

26) Do you make any allowances if it's a first season bull as regards bull/cow ratio?

____

27) Do you test the semen of the bull at purchase? _________________________

28) What do you test the semen for?

Semen concentration and quality	
Evidence of disease/Specify (e.g. IBR, BVDV)	

29) How do you know your bull is working in the breeding season?

Scanning carried out during the breeding season	
By recording services and not having cows return to heat	
I don't	

30) What breed of bull did you use in 2009 and 2010?

____

31) What is your source of replacement heifers?

Home bred	
Purchased	
Both	

32) Do you buy in replacement heifers as maidens or as in-calf?

	2009	2010
Maiden		
In-calf heifers		

33) What age do you normally put heifers in-calf? _______________

34) If already in calf at purchase what age are they at calving down? ________________

35) Do you normally use artificial insemination on heifers or put them to a bull? ______________________________

36) In 2009 and 2010 what did you do? How many by either method?

	A.I.	Stock bull
2009		
2010		

37) In 2009 and 2010 how many cows were served by bull or put to A.I.?

	A.I.	Stock bull
2009		
2010		

38) Do you calve your heifers at the start of the breeding season?

____

39) When in relation to the start of the breeding season do you serve your heifers? ____________________________

40) What breed of bull is used on heifers? _____________________________

41) If artificial insemination is carried out who performs it?

Technician		Farmer		Both	

42) If the farmer performs it when did she/he first train? ________________

43) When was the most recent refresher course done? ____________

44) Is heat detection carried out on farm? ______________________

45) If yes, then how many times a day is it performed?

	1	2	3	4	Greater than 4
Times/ day					

46) Are heat detection aids used? ________________________

What aids are used?

	Cows	Heifers
Tail paint		
Vasectomised bull		
Scratch cards		
If other methods specify		

47) Are cows scanned for pregnancy? _______________________

48) Are heifers scanned for pregnancy? ___________________

49) If not are they done manually? Heifers ___________ Cows ___________

50) Who performs the pregnancy diagnosis? ____________________

51) Are all breeding females done as a group or in groups? ___________________

52) When are they done in relation to the breeding season?

____

53) Did you use any synchronisation programmes on the cows and heifers in 2009 and 2010? Give details

54) Do you use any intervention for getting late calvers back cycling earlier?

____

55) Give details of this intervention.

Biosecurity:

1) Total number of farm staff ________________________

	Amount
Family members	
Full-time staff that are not family members	
Casual labour	

2) Do any staff have regular contact with other livestock?

Yes		No	

3) What visitors have direct contact with your stock?

4) What type of farms bound your land?

Cattle	
Sheep	
Tillage	
Other	

5) Have you had animals from neighbouring farms enter your land in the last 5 years?

____

6) Have your animals escaped into neighbouring farms in the last 5 years?

____

7) Is your land all in one block, if not then how many parcels including the main block? ____________________

8) What type of farms bound these parcels?

	Parcel 2	Parcel 3	Parcel 4
Cattle			
Sheep			
Tillage			
Other			

9) Please detail stock purchases over the last 3 years.

10) Are bought in animals isolated on arrival? ________________________

11) Are they isolated for at least 30 days at a distance of at least 3 metres with no mixing of urine and dung? ____________________

12) If bought in animals are tested for biosecure diseases (such as IBR, BVDV etc.) please specify what they are tested for?

13) Give details of vaccine/treatment regimes for bought in stock.

14) Is there a foot dip in use for visitors? ________________________

15) How frequently are the contents replenished?

____

16) Are the contents replenished in accordance with manufacturer's instructions?

____

17) Are regular checks made to the boundary fencing? _________________

18) Is it stockproof? Quantify how often.

A		B	Every(how many)			
Yes			Day		Month	
No			Week		Year	

19) Is there a 3 metre divide between your stock and the stock of the farmer next door?

Yes	
No	

20) If slurry is spread how soon do animals enter that field afterwards on average?

____

21) Are livestock trailers cleaned and disinfected if used off farm?

Yes	
No	

22) Do the following visitors clean and disinfect themselves (boots, equipment and overalls) on entering your farm?

	Yes	no
Inseminator		
Vet.		
Agricultural advisor		
Hoof Trimmer		
Scanner		
Others		

23) Are animals co-grazed with other species such as sheep? Give details.

24) Do you keep dogs and cats on the farm?

Yes	
No	

25) Have you seen any of the following on your farm in 2009 and 2010?

	2009	2010
Foxes		
Badgers		
Deer		

26) Are there any rivers or streams coursing through your farm?

Yes	
No	

27) If yes, are there farms upstream from you?

Yes	
No	
Don't know	

28) What animals have access to these water courses?

____
