# Supplementary material for: Herd health status and management practices on 16 Irish suckler beef farms
Source: Ir Vet J. 2013 Nov 6;66(1):21. doi: 10.1186/2046-0481-66-21 (PMC3903451; doi:10.1186/2046-0481-66-21)
Supplement: Additional file 2 — BETTER farm animal health veterinary questionnaire. [file 2046-0481-66-21-S2.pdf]

Teagasc/IFJ BETTER farm animal health veterinary questionnaire

Date:

Farmer:

Veterinary clinician:

Address:

Phone number:

Mobile:

Email:

Fax:

Service History:

1) How long has this practice provided a veterinary service to this farm? ___________

2) Was your practice solely responsible for all clinical work provided to this farm in 2009 and 2010?

	2009	2010
Yes		
No		

3) How many vets from the practice do the clinical work on the farm?

One		Three	
Two		More than three	

4) Apart from herd/private TB/Brucellosis work, how many visits on average were carried out in 2009 and 2010 to the farm?

2009 ______________ 2010 ________________

5) Do you know of any other veterinary practice providing a service to your client in 2009 or 2010? _______________________

6) Do you know whether or not your client used a service other than the one you provide to carry out reproductive work in 2009 _____________ or 2010 ________________?

Herd health planning

1) Has a formal herd health plan been designed for this farm?

Yes ________ No _________

2a) If there has been, what aspects does it cover?

2b) If not, which of the following do you routinely advise your client on?

	2009	2010
Parasite control		
Vaccination protocols		
Fertility		
Nutrition/minerals		
Calf rearing/health		
Management of replacement heifers		
Biosecurity		
Post-calving management		
Pre-calving management		
Protocol for bull/Replacement purchases		
Management/control of lameness		
Other advice(please specify)		

3) Did you carry out any of the following procedures on the farm in 2009 and 2010? For each year please enter the number of times for each procedure and the results if applicable.

	2009		2010	
	Times	Results	Times	Results
Disbudding calves				
Dehorning (>1 month)				
Castrating				
Regular Pregnancy diagnosis				
Body condition scoring				
Regular hoof trimming				
Blood monitoring for minerals				
Faecal testing for gut worms				
Faecal testing for lungworm				
Faecal testing for liver/rumen fluke				
Monitoring for BVDV				
Monitoring for IBR				
Monitoring for Johne's				
Monitoring for leptospirosis				
Nasal swabs for respiratory viruses				
Post mortem examinations				

4) What were the biggest on farm issues from your point of view in 2009 and 2010?

	2009	2010	Action/Treatment undertaken + duration
Calf scour			
Joint ill			
Navel ill			
Septicaemia			
Ill-thrift in calves			
Pneumonia in calves			
Meningitis			
CCN			
Coccidiosis			
Clostridial disease			
BRD in older groups(viral/bacterial)			
Fog fever			
Pink eye/New forest eye			
Gut/stomach worms			
Liver fluke , Rumen fluke			
Lungworm			
Lice			
Mange			
Other parasites e.g. Babesia			
Mineral/Vit. deficiencies			
Any toxicities			
Infertility			
Abortion			
Lameness			
Calving oversize			
Slow/Delayed calvings			
Stillborn/weak calves			
Congenitally deformed calves			
Retained cleansings			
Ill-thrift in older stock			
Acidosis/grain overload			
Chronic diarrhoea			
Hypomagnesaemia/Tetany			
Dry period mastitis			
Mastitis during lactation			
On farm sudden deaths			
Deaths due to injury			
Other issues(specify)			
